# Trends of Tourette Syndrome in children from 2011 to 2021: A bibliometric analysis

**DOI:** 10.3389/fnbeh.2022.991805

**Published:** 2022-11-17

**Authors:** Cuiling Yang, Jie Zhang, Qiong Zhao, Jingjin Zhang, Jiang Zhou, Li Wang

**Affiliations:** ^1^Department of Pediatrics, Hospital of Chengdu University of Traditional Chinese Medicine, Chengdu, China; ^2^Library, Chengdu University of Traditional Chinese Medicine, Chengdu, China; ^3^Department of Pediatric Orthopedics, Chengdu No.1 Orthopedics Hospital, Chengdu, China

**Keywords:** Tourette syndrome, children, Web of Science, CiteSpace, data visualization

## Abstract

**Objective:**

Analyze the research status of Tourette Syndrome (TS) in children by CiteSpace and determine the current research hotspots and frontiers.

**Materials and methods:**

We chose publications indexed in the Web of Science Core Collection (WoSCC) database for studies related to TS in children from 2011 to 2021. We built online cooperation maps of countries/regions, institutions, authors, journals, references, and keywords by CiteSpace, and identified hotspots and frontiers of study for children’s TS.

**Results:**

A total of 1,232 publications about TS in children were downloaded from the WoSCC. The USA (414) was the country with the highest rate of production, and University College London (87) was the institution that had the highest publication rate. Andrea Eugenio Cavanna was the most prolific author (39 papers). There was inactive cooperation between institutions, countries/regions, and authors. The Journal of *European Child & Adolescent Psychiatry* was the most active journal. Hot topics focused on epidemiology, comorbidities, deep brain stimulation, behavioral therapy, basal ganglia, pharmacological treatment, and risk factors of TS in children.

**Conclusion:**

According to the CiteSpace results, this study found that authors, countries/regions, and institutions were not actively working together. Current research hotspots mainly consist of epidemiology, comorbidities, deep brain stimulation, behavior therapy, and basal ganglia. The main research trends include comorbidities, pharmacological treatment, and risk factors. Therefore, international cooperation should be strengthened in the future, and it should be mindful of the psychiatric comorbidities of TS, the choice of intervention measures, and early warning of risk factors.

## Introduction

Tourette Syndrome (TS) is a neuropsychiatric disorder characterized by chronic motor and/or vocal tics lasting more than 1 year (Rae et al., 2018). These symptoms often occur in about 1% of children of school age, which interfere with children’s academic performance and daily life activities (Mufford et al., 2019). However, TS is often undiagnosed or misdiagnosed (Eapen et al., 2016). TS often has frequent comorbidities with obsessive compulsive disorder (OCD), attention deficit/hyperactivity disorder (ADHD), or autism (Set and Warner, 2021; Eapen and Usherwood, 2022). In recent years, people have become more aware of the consequences, results, and burdens of TS. However, systematic research on worldwide research trends and hotspots is lacking. It is necessary to review the current research status and provide a reference for future research. This study aims to systematically compile all available literature on TS from 2011 to 2021, review the state of the field, identify emerging trends and hot spots, and investigate the potential issues in this field to advance future research and clinical application.

Bibliometrics is a method of using metrics (indicators) to evaluate research performance (Zhong et al., 2020; Shi et al., 2021). It can quantitatively and qualitatively measure the structure, growth, and trends of knowledge about a subject and is now used in a wide range of fields (Chen et al., 2021). Here, CiteSpace will be utilized for bibliometrics as well as visual analysis (Du et al., 2021). This is the first time visual analysis has been done with CiteSpace in the field of TS. We concentrated mainly on the network and cluster analysis of countries/regions, institutions, and co-authors, cited references, and keywords, and explored the hotspots and trends of TS.

## Materials and methods

### Data source and search strategy

For this study, we chose publications indexed in the Web of Science Core Collection (WoSCC) database as the data source. The WoSCC database is commonly utilized in bibliometric analysis since it strictly evaluates articles, ensuring high-quality literature. The WoSCC database, on the other hand, is constantly and dynamically updated and can give you the most important, relevant, and reliable information. Because of this, this database was good for our research.

**FIGURE 1:**
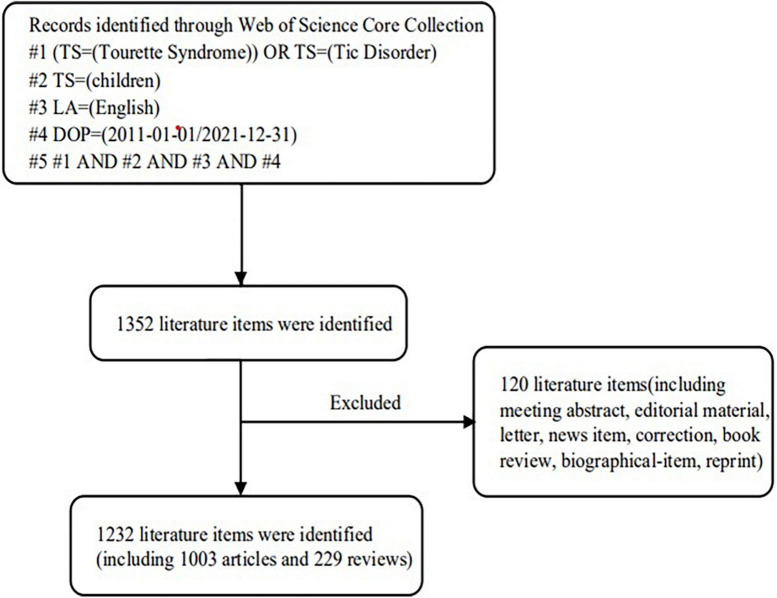
Frame flow diagram of search strategy.

These terms were included in the topic and searched for: (“Tourette Syndrome” OR “Tic Disorder”) AND (“children”) AND Language = English. Document types only contain articles and reviews. We searched the WoSCC database exhaustively for relevant data released from 2011 to 2021. Figure 1 depicts the detailed search process.

### Analysis tool

We used CiteSpace 5.8 R3 and Excel 2019 to analyze the data in this study. Dr. Chaomei Chen, a scholar at Drexel University in the United States, created CiteSpace, a bibliometric analysis visualization software. A “Plain Text” form of each record’s “Full Record and Cited References” was retrieved from WoSCC and imported into CiteSpace. Our analysis included the annual publication volume, journals, countries/regions, institutions, authors, references, and keywords. By analyzing the number and growth trend of papers published each year, investigating author/institutional/country collaboration networks, detecting co-cited references, and keeping track of keywords with high citation bursts over time, the research frontiers and emerging trends of TS in children were identified. As the circle grows larger in CiteSpace, it indicates the growing number of papers that have been published. Meanwhile, the shorter the distance between the two circles, the closer the cooperation between the two circles. Meanwhile, nodes with a high betweenness centrality (BC ≥ 0.1) are frequently highlighted with purple rings, which is an important parameter for assessing the scientific value of the nodes. CiteSpace provides two indexes based on network structure and clustering clarity to evaluate graph drawing: modularity (Q) and silhouette (S). Generally speaking, Q is generally within the interval [0.1], and Q > 0.3 means that the community structure is significant. If S > 0.5, clustering is generally considered reasonable. When S > 0.7, clustering is efficient and convincing.

### Quality control

All data downloads and literature searches were done on February 15, 2022, so that the database update wouldn’t cause any bias. Methods for controlling research quality: (1) two researchers worked independently on the data analysis, and any disagreements were resolved through discussion or by enlisting the assistance of outside experts. (2) the final results of the knowledge map would be confirmed by experts in related fields to verify the guiding significance of the research conclusions for clinical practice.

## Results

### Analysis of publication years

**FIGURE 2:**
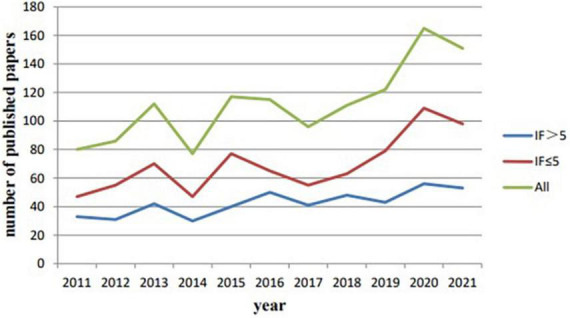
Annual trend chart of publications related to Tourette Syndrome (TS) in children research from 2011 to 2021.

Through a preliminary search of literature on TS in children, 1,232 publications were retrieved from the WoSCC database (Figure 1). In order to better understand the literature distribution, we divided all publications into >5 and ≤5 groups according to the JCR impact factor (IF) (2021). There were 467 articles (37.91%) with IF > 5, and 765 articles (62.09%) with IF ≤ 5. Figure 2 shows the trend of publications in the year after grouping according to IF. It can be seen that the publications with IF ≤ 5 show a fluctuating growth trend, which is basically consistent with the trend of the overall number of publications, while the publications with IF > 5 also shows a growing trend. When the overall number of publications decreases, the number of literature also decreases. At the same time, according to Figure 2, there has been a fluctuating increase in the number of annual publications related to TS in children, from 80 in 2011 to 151 in 2021. This field had the fewest number of articles in 2014, only 77. However, there was rapid growth in 2017–2020, which shows that the field has received more and more attention during this period.

### Analysis of countries/regions

**FIGURE 3:**
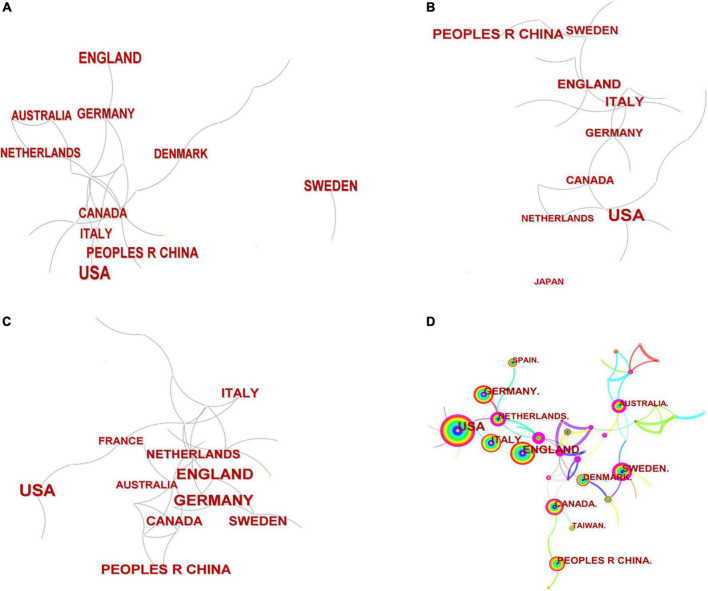
A country/region cooperation map related to Tourette Syndrome (TS) in children research from 2019 **(A)**, 2020 **(B)**, 2021 **(C)** and from 2011 to 2021 **(D)**.

The network of national partnerships for TS in children’s research is shown in Figure 3, which covers national cooperation maps for all literature in the years before and after COVID-19 (2019, 2020, and 2021), as well as during the study period. Table 1 provides a list of the top 5 contributing nations, each of which has contributed 965 articles and accounts for 78.33% of publications. The top contributor was the USA (33.60%), followed by England (15.75%), Italy (10.06%), Germany (9.82%), and China (9.09%). The analysis revealed that the top five countries/regions in terms of centrality were Hungary (0.51), Scotland (0.42), France (0.41), the Netherlands (0.31), and Australia (0.24). According to Figure 3, there was no significant difference in national cooperation structure before and after COVID-19. Meanwhile, the graph of country-based research networks shows a lower density, which suggests that research teams are relatively independent and highlights the need for more collaboration.

**TABLE 1:** The top 10 countries/regions related to Tourette Syndrome (TS) in children research from 2011 to 2021.

Ranking	Country/Region	Frequency	Ranking	Country/Region	Centrality
1	USA	414	1	Hungary	0.51
2	England	194	2	Scotland	0.42
3	Italy	124	3	France	0.41
4	Germany	121	4	Netherlands	0.31
5	China	112	5	Australia	0.24

### Analysis of institutions

**FIGURE 4:**
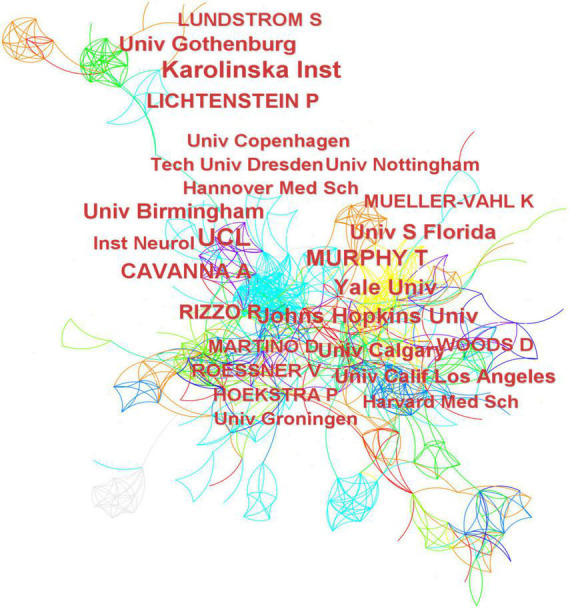
Institutions cooperation and co-authors map related to Tourette Syndrome (TS) in children research from 2011 to 2021.

Figure 4 shows the main network of institutional collaborations and co-author related to children with TS, with the University College London in England being the most productive institution in this field. Table 2 illustrates the top 10 most prolific institutes in this research field, contributing 494 articles (40.10%), including University College London (7.06%), Karolinska Institutet (6.17%), Yale University (4.30%), University of Birmingham (3.98%), and Johns Hopkins University (3.73%), four of which are from the USA. Karolinska Institutet (0.13), Johns Hopkins University (0.12), and University College London (0.1) had the highest centrality rankings. Centrality is low for all institutions, which indicates poor collaboration among institutions.

**TABLE 2:** The top 10 Institutions related to Tourette Syndrome (TS) in children research from 2011 to 2021.

Ranking	Institution	Frequency	Country/Region	Centrality
1	University College London	87	England	0.1
2	Karolinska Institutet	76	Sweden	0.13
3	Yale University	53	USA	0
4	University of Birmingham	49	England	0.09
5	Johns Hopkins University	46	USA	0.12
6	University of Gothenburg	43	Sweden	0.05
7	University of South Florida	38	USA	0.01
8	University of Calgary	37	Canada	0.01
9	University of California Los Angeles	34	USA	0.04
10	Technical University Dresden	31	Germany	0.02

### Analysis of co-authors

The top 10 authors were responsible for 291 papers (23.62%) (Table 3). Andrea Eugenio Cavanna was the author who produced the most works (3.17%), followed by Paul Lichtenstein (2.60%), Douglas W Woods (2.44%), and Sebastian Lundström (2.35%). In terms of centrality, Veit Roessner (0.40), Renata Rizzo (0.37), and Andrea Eugenio Cavanna (0.13) had the highest centrality rankings, while the centerlines of the rest of the prolific authors were less than 0.1. This indicates that some authors who publish a large number of publications tend to maintain stable cooperative partnerships with other authors.

**TABLE 3:** The top 10 authors related to Tourette Syndrome (TS) in children research from 2011 to 2021.

Ranking	Author	Frequency	Centrality	Year of first article
1	Andrea Eugenio Cavanna	39	0.13	2011
2	Paul Lichtenstein	32	0.01	2013
3	Douglas W Woods	30	0.08	2012
4	Sebastian Lundström	29	0.05	2013
5	Renata Rizzo	29	0.37	2011
6	Veit Roessner	29	0.40	2011
7	Davide Martino	27	0.01	2011
8	Tanya K Murphy	26	0.10	2011
9	James F Leckman	26	0.01	2011
10	Eric A Storch	24	0.00	2011

### Analysis of journals and cited journals

The number of journals that published 1,232 articles on TS in children was 396. Table 4 lists the 10 most active journals, and most of these journals’ publishers are based in the USA or the Netherlands. The majority of the publications were published in the Journal of *European Child & Adolescent Psychiatry* and the Journal of *Child and Adolescent Psychopharmacology*, followed by *Psychiatry Research* and the *Journal of Child Neurology*.

**TABLE 4:** The top 10 journals with the highest frequency related to Tourette Syndrome (TS) in children research from 2011 to 2021.

Ranking	Journal	Frequency	Country/Region
1	*European Child & Adolescent Psychiatry*	48	Germany
2	*Journal of Child and Adolescent Psychopharmacology*	47	USA
3	*Psychiatry Research*	36	Netherlands
4	*Journal of Child Neurology*	32	USA
5	*Frontiers in Psychiatry*	31	Sweden
6	*Journal of Child Psychology and Psychiatry*	28	England
7	*Movement Disorders*	24	USA
8	*Pediatric Neurology*	20	USA
9	*Journal of the American Academy of Child and Adolescent Psychiatry*	19	Netherlands
10	*Frontiers in Neurology*	17	Switzerland

Co-citation of journals can reflect the correlation between journals. Table 5 shows the 10 most cited journals. The *Journal of the American Academy of Child and Adolescent Psychiatry* receives the most citations (898), followed by the *American Journal of Psychiatry* (645), and the *Archives of General Psychiatry* (580). In terms of centrality, the centerlines of the top 10 cited journals are mostly ≥0.1, suggesting that the cooperation of journals is closer.

**TABLE 5:** The top 10 co-cited journals with the highest frequency related to Tourette Syndrome (TS) in children research from 2011 to 2021.

Ranking	Cited journal	Frequency	Centrality	Country/Region
1	*Journal of the American Academy of Child And Adolescent Psychiatry.*	898	0.12	Netherlands
2	*American Journal of Psychiatry*	645	0.07	USA
3	*Archives of General Psychiatry*	580	0.16	USA
4	*Neurology*	543	0.13	USA
5	*Movement Disorders*	538	0.10	USA
6	*European Child & Adolescent Psychiatry*	524	0.12	Germany
7	*Journal of Child Psychology and Psychiatry*	469	0.12	England
8	*Biological Psychiatry*	438	0.15	Netherlands
9	*Developmental Medicine & Child Neurology*	411	0.01	USA
10	*Pediatrics*	408	0.05	USA

### Analysis of co-cited references

A total of 31,968 references were extracted from 1,232 articles for citation analysis. The time span is from 2011 to 2021, and the time slice is one. After selecting references as node types for statistical analysis, 50 items that are most frequently cited or appear in each time slice are selected to build a cited literature network diagram made up of 338 nodes and 1,940 links. Table 6 lists the top 10 studies related to TS in children, which have been cited more than 900 times. An analysis in terms of co-citation counts and centrality (Table 6) revealed that the data on this topic over the past decade is generally in the form of (1) clinical guidelines, (2) controlled trials, and (3) epidemiological and prevalence studies.

**TABLE 6:** The top 10 co-cited references sorted with the highest citations related to Tourette Syndrome (TS) in children research from 2011 to 2021.

Ranking	Co-cited references	Citation	Centrality	Representative author (Publication year)
1	Diagnostic and Statistical Manual of Mental Disorders	132	0.01	American Psychiatric Association (2013)
2	Behavior therapy for children with Tourette disorder: a randomized controlled trial	130	0.18	Piacentini et al. (2010)
3	European clinical guidelines for Tourette syndrome and other tic disorders. Part II: pharmacological treatment	115	0.17	Roessner et al. (2011)
4	Lifetime Prevalence, Age of Risk, and Genetic Relationships of Comorbid Psychiatric Disorders in Tourette Syndrome	106	0.07	Hirschtritt et al. (2015)
5	Prevalence of Tic Disorders: A Systematic Review and Meta-Analysis	99	0.05	Knight et al. (2012)
6	European clinical guidelines for Tourette Syndrome and other tic disorders. Part III: behavioural and psychosocial interventions	83	0.06	Verdellen et al. (2011)
7	Clinical course of Tourette syndrome	60	0.01	Bloch and Leckman (2009)
8	The international prevalence, epidemiology, and clinical phenomenology of Tourette syndrome: A cross-cultural perspective	59	0.05	Robertson et al. (2009)
9	The prevalence and epidemiology of Gilles de la Tourette syndrome Part 1: The epidemiological and prevalence studies	57	0.06	Robertson (2008)
10	Quality of life in young people with Tourette syndrome: a controlled study	54	0.05	Eddy et al. (2011)

**FIGURE 5:**
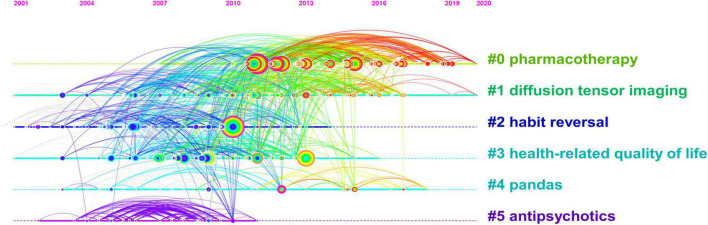
A reference co-citation map related to Tourette Syndrome (TS) in children research from 2011 to 2021.

To evaluate the nominal terms derived from the article keyword list for cluster names, to acquire the crucial data from the cited references, and to investigate the research models and new trends in the knowledge system, we used the logarithmic likelihood ratio (LLR) algorithm. Figure 5 shows the timeline view of the reference co-citation network. Six clusters with a modularity value of 0.5909 and a silhouette of 0.8825 are generated, which means that our cluster results are highly reliable, reasonable, and meaningful. The largest group was cluster # 0, “pharmacotherapy,” which was painted in cold colors to signify that it would be a research hotspot in the near future. The second is cluster # 1, “diffusion tensor imaging,” which has always been a hot spot in the study of this topic.

### Analysis of co-occurrence keywords and cluster

**FIGURE 6:**
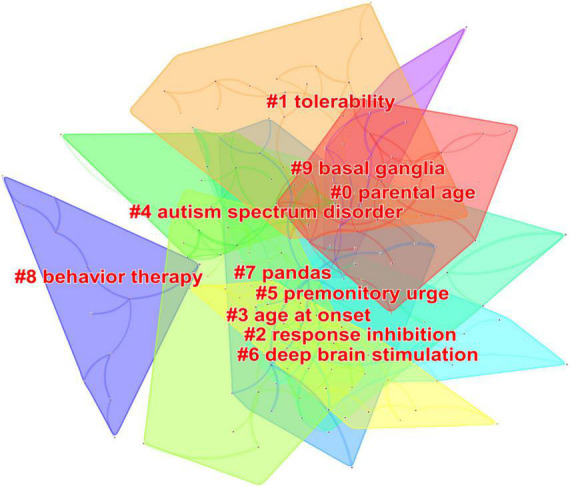
Keywords cluster analysis co-occurrence map related to Tourette Syndrome (TS) in children research from 2011 to 2021.

The hot topics in a research field can be identified by analyzing the high-frequency keywords, while the status and influence of the associated study topic are indicated by the greater central keywords. A network of keyword co-occurrences consisting of 198 nodes and 1,694 linkages was produced (Table 7). Table 7 shows that the top 10 high-frequency keywords were: Tourette syndrome, children, deficit hyperactivity disorder, health-related quality of life, obsessive compulsive disorder, epidemiology, spectrum disorder, behavior therapy, controlled trial, and depression. The top 10 high-centrality keywords were: neuropsychiatric disorder, deficit hyperactivity disorder, deep brain stimulation, basal ganglia, obsessive compulsive disorder, spectrum disorder, tic severity, symptom, schizophrenia, and diagnosis. In addition, ten clusters were found, and each had a silhouette value that was greater than 0.8, indicating that the findings were valid and meaningful (Figure 6 and Table 8).

**TABLE 7:** The top 10 keywords related to Tourette Syndrome (TS) in children research from 2011 to 2021.

Ranking	Frequency	Keyword	Ranking	Centrality	Keyword
1	799	Tourette syndrome	1	0.13	neuropsychiatric disorder
2	733	children	2	0.12	deficit hyperactivity disorder
3	300	deficit hyperactivity disorder	3	0.12	deep brain stimulation
4	274	health-related quality of life	4	0.11	basal ganglia
5	259	obsessive compulsive disorder	5	0.10	obsessive compulsive disorder
6	240	epidemiology	6	0.10	spectrum disorder
7	222	spectrum disorder	7	0.08	tic severity
8	148	behavior therapy	8	0.07	symptom
9	141	controlled trial	9	0.07	schizophrenia
10	132	depression	10	0.07	diagnosis

**TABLE 8:** Keywords cluster analysis.

Cluster ID	Silhouette	Mean (Year)	Label (LLR)	Included keywords (Top five)
0	0.942	2012	Parental age	Tourette syndrome; conduct disorder; neurodevelopmental disorders; tics; infection
1	0.932	2013	Tolerability	premonitory urges; schizophrenia; sensorimotor gating; comorbidity; inhibitory control
2	0.86	2013	Response inhibition	inhibition; connectivity; volume; enhanced cognitive control; response inhibition
3	0.858	2012	Age at onset	tolerability; double blind; aripiprazole; controlled trial; open label
4	0.847	2014	Autism spectrum disorder	deep brain stimulation; obsessive compulsive disorder; management; quality; randomized controlled trial
5	1	2013	Premonitory urge	obsessive-compulsive disorder; reliability; scale; validity; cluster analysis
6	0.992	2014	Deep brain stimulation	behavior therapy; cbit; guideline; treatment evaluation; supportive psychotherapy
7	0.953	2012	Pandas	movement disorders; movement disorder; statistical learning; headache; neuropsychology
8	0.899	2012	Behavior therapy	onset; psychiatric disorder; obsession; compulsion; pandas
9	1	2014	Basal ganglia	adhd; tourette syndrome; attention-deficit hyperactivity disorder; hyperactivity disorder; deficit hyperactivity disorder

### Analysis of keywords with citation bursts

**FIGURE 7:**
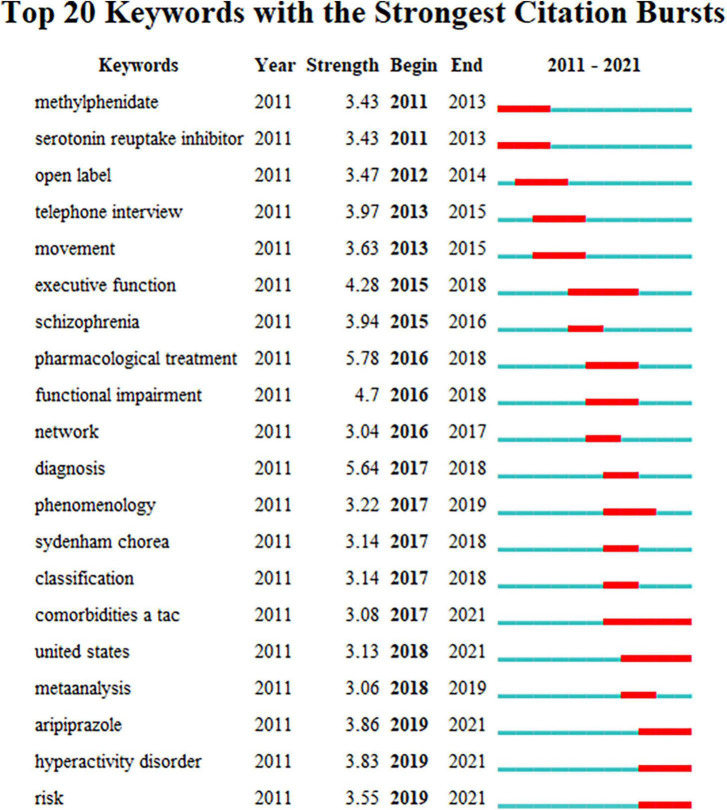
Top 20 keywords with the strongest citation bursts.

Burst keywords are used to record keywords that have undergone significant change in a short period of time and are automatically created by the software according to the keywords in the list. It is widely regarded as another important research hotspot or an indicator of upcoming developments. The keyword burst period is shown by the red line, and the time period is shown by the blue line. The top 20 keywords with the most powerful citation bursts are displayed in Figure 7. From 2011 to 2021, pharmacological treatment had the highest burst strength (5.78).

## Discussion

As far as we know, this is the first bibliometric study on TS to review the research status and to reveal the associated research hotspots and frontiers. We searched for data from 1,232 studies published between 2011 and 2021. CiteSpace and Excel were employed for the analysis, and descriptive statistics, symbiosis, and cluster analysis were the methods that were applied to the datasets that were obtained from these studies.

### General information

From 2011 to 2021, the growth trend of obtaining publications related to TS from WoSCC fluctuated over time but showed an overall increasing trend, reflecting the increasing importance of TS in the field of neurological research in children. From the distribution of IF, the literature distribution we obtained is reasonable and can be studied. According to Figure 2, we find that the number of publications decreased in 2014 and 2017. We checked the data over the years and excluded political and economic factors. In May 2013, the American Psychiatric Association (APA) launched the latest version of the American Classification and Diagnosis of Mental Disorders (DSM-5), and in 2017 the American Academy of Neurology (AAN) formulated *the Practice Guideline: The treatment of tics in people with Tourette syndrome and chronic tic disorders*, so it is considered that the decrease in the number of publications may be related to these factors.

The USA is in a leading position in this field, reflecting its leading position in TS research. Developed countries/regions are the main force behind TS research. This trend shows that the development of scientific research is related to the mature medical research environment and economic conditions of the country. At the same time, the large population is also an important factor affecting the number of publications. Being the sole developing nation in the top five, China has recently achieved significant advancements in TS. Additionally, in terms of national cooperation, Hungary, Scotland, France, and the Netherlands cooperate more closely with other countries/regions, which may have something to do with each country’s geographical location. It is found that the COVID-19 has no significant impact on the national cooperation with TS. In addition, it has been observed that there is less cooperation among countries/regions with more contributions, as can be seen from their relatively low centrality. We suggest that international cooperation, especially with leading countries/regions in this field, should be strengthened, which will accelerate global research progress in TS.

In terms of institutions, they are mostly located in the USA and England. University College London, Karolinska Institutet, and Johns Hopkins University are prolific and highly centralized institutions, which shows that they are important institutions for studying TS and working closely with other institutions. However, Italy and China are two of the countries/regions that rank among the top five in terms of the total number of publications, but there are no institutions in the top 10. This may indicate that there are relatively few institutions conducting research in this area in the country. Choosing a suitable partner institution is conducive to better TS research.

Although Andrea Eugenio Cavanna is the most prolific writer (Pringsheim et al., 2019b), the frequently cited literary works were not written by him. And among the top 10 prolific writers, only four writers’ centrality ≥ 0.1. It is worth noting that most of the global TS researchers for children are from Europe and the United States, and this result is consistent with the cooperative relationship between the previous countries/regions. Therefore, children’s TS research still needs to strengthen communication and cooperation among global researchers. In addition, most of the cooperative relationships between the authors are closely related to their respective institutions, indicating that the authors are more inclined to work with stable cooperative teams.

The top 10 journals published 302 papers, accounting for 24.51% of the total, indicating that the top 10 journals are more interested in TS research. The major journals in the fields of pediatrics and neurology, which have had a great impact on pediatricians and neurologists around the world and have impacted the direction of research in the related fields of science, are the most often published journals in TS research. These results can help scholars choose more suitable journals when submitting papers in related fields.

### Research hotspots

References can reflect important insights into currently known information about a certain topic. Co-citation analysis can reveal the knowledge structure of a research field by analyzing the clustering and key nodes in the co-citation network. The top 10 co-cited references indicated that scholars are more focused on the guidelines for clinical diagnosis and treatment, controlled trials, and epidemiology of TS. Especially noteworthy, the first reference with the greatest rate of co-citation was the *Diagnostic and Statistical Manual of Mental Disorders* proposed by the American Psychiatric Association (American Psychiatric Association, 2013), which proposed the diagnostic criteria, evaluation methods, and treatment of TS. A randomized controlled trial of behavioral therapy was the top reference by centrality in children with TS, indicating that comprehensive behavioral intervention can improve the symptom severity among children with TS. In addition, according to the timeline view of the network for reference co-citation, it is found that pharmacotherapy is the focus of TS research, but the attention of antiphychotics has decreased in the past decade, which is closely related to the research progress of TS. At the same time, diffusion tensor imaging can assist in the study of the pathogenesis and efficacy of TS.

Keywords are the high-level summary of the core content of the research. Keyword co-occurrence analysis can help researchers identify hot topics within a specific subject matter. Co-occurrence keyword and cluster analysis results demonstrate that the principal hot subjects for the present study include epidemiology, comorbidities, deep brain stimulation, behavior therapy, and basal ganglia. Burst keywords can indicate the research frontiers, reveal the potential studies, and thus predict the future research trend. The results of burst keywords suggest that pharmaceutical treatment, comorbidities, and risk factors are the main research trends of TS.

#### Epidemiology

Epidemiological studies, including the distribution characteristics, prevalence, and influencing factors of the disease population, are of great significance for the prevention and treatment of diseases. Childhood-onset primary tic disorder is common (American Psychiatric Association, 2013). It is found that TS is more likely to influence boys than girls (Ueda and Black, 2021). According to the 2011–2012 National Survey of Children’s Health (NSCH), 0.28% of children in the United States are suffering from TS (Bitsko et al., 2014). A Statistics Canada survey shows that the prevalence rate of TS among Canadian teenagers (12–17 years old) is 0.33% (Yang et al., 2016). The incidence of TS has seldom been evaluated in Asia. It is suggested that countries/regions around the world conduct epidemiological studies on TS in order to better comprehend TS’s nature, prevalence, and risk factors.

#### Comorbidities

TS is commonly comorbid with a variety of mental and/or behavioral disorders, such as ADHD, OCD, autism spectrum disorder (ASD), depression, anxiety disorder, sleep disorders, and self-injurious behavior (Stafford and Cavanna, 2020; Keenan et al., 2021; Jones et al., 2022). About 90% of the patients have psychiatric comorbidities, of which ADHD, OCD, and ASD are the most common, followed by other diseases (Hirschtritt et al., 2015; Robertson et al., 2017). This is probably due to the overlap in neurobiology and pathophysiology and genetic inheritance of these disorders compared to TS (Mathews and Grados, 2011; Grotzinger et al., 2022). Comorbidities have a significant detrimental impact on patients’ quality of life (Robertson et al., 2015; Deeb et al., 2019). Comorbidities with ADHD can lead to behavioral disorders such as aggressive behavior, destructive behavior, poor academic performance and social adaptability, and executive function problems (Termine et al., 2016; Openneer et al., 2020). Patients with TS and OCD have more desires and impulses to repeat checking, reordering, or fixing procedures, resulting in severe clinical distress or social or occupational dysfunction (Bellato et al., 2021). Trichotillomania may also be seen in patients with TS and OCD (Lamothe et al., 2020). In addition, the comorbidities of TS can contribute to these feelings involving anxiety, tension, stress, and frustration (Leisman and Sheldon, 2022). The comorbidities of TS increase the complexity and severity of TS, have an impact on how well children learn, socialize, and develop their personalities and psychological qualities, and make it more difficult to diagnose, treatment, and the prognosis of the disease. At times, the comorbidities are more problematic and will need more intervention than the tics themselves. How would you screen for comorbidities in a patient with possible TS? And truthfully, it is a subject worth considering.

#### Deep brain stimulation

Deep brain stimulation (DBS) is an established treatment for neurological and psychiatric disorders. For patients with severe drug-refractory TS, DBS is a promising treatment option (Kantzanou et al., 2021). One study indicated that DBS mainly acts on a variety of regions and structures located within the network of cortico-striato-pallido-thalamo-cortical tissue (Akbarian-Tefaghi et al., 2017). Another study reported that DBS produced a mean 50% improvement in overall tic severity by the total Yale Global Tic Severity Scale (YGTSS) score (Dowd et al., 2018). Kara et al. found that several months of DBS treatment can effectively improve TS and OCB (Johnson et al., 2019). A recent systematic review and meta-analysis reported that DBS for TS had an overall improvement of 53% (YGTSS score) (Baldermann et al., 2016). In a study including 185 patients from 10 countries/regions in the International Deep Brain Stimulation Database and Registry, the mean YGTSS score improved 45.1% at 1 year after DBS implantation (Martinez-Ramirez et al., 2018). However, the safety of DBS still needs attention, including visual disturbances, dysarthria, paresthesia, intracranial hemorrhage, and infection (Schrock et al., 2015; Martinez-Ramirez et al., 2018; Smeets et al., 2018). The mechanisms by which DBS works are not fully understood, but functional magnetic resonance imaging (fMRI) may help to fill this knowledge gap (Loh et al., 2022). According to an analysis of fMRI, the subthalamic nucleus was the area of the brain that was most frequently stimulated (Loh et al., 2022). Considering the higher risk of complications after DBS, we figure the sensible thing to do is to better understand the feasibility, safety, and clinical effectiveness of DBS in the treatment of severe-refractory TS.

#### Behavior therapy

Behavioral therapy is the first-line treatment for TS, which can reduce tic symptoms and comorbidities and improve social functioning (Pringsheim et al., 2019b; Andren et al., 2022), including habit reversal training, exposure and response prevention, relaxation training, positive reinforcement, self-monitoring, regression exercise, and the most frequently employed is the Comprehensive Behavioral Intervention for Tics (CBIT) (Espil et al., 2021). The AAN suggests that CBIT should be considered as the initial treatment option for TS, which reduces tics by training patients and teaching them specific behavioral strategies (Pringsheim et al., 2019b). Flint et al. found that behavioral therapy achieved remission (67%) at follow-up on the YGTSS, and tic severity decreased significantly across the sample (*n* = 126) (Espil et al., 2021). A meta-analysis and literature review showed that CBIT can significantly reduce the total score of tic disorder and the score of motor tics, but not the score of vocal tics (Shou et al., 2022). Behavioral therapy is much safer than pharmacological treatment because the core of behavioral therapy is the cognitive function of children in the recognition and control of impulses, so it is only effective for older children with TS, and the implementation environment and compliance requirements are high, for many countries/regions still face great challenges. By far the most common form of behavioral therapy is to deliver therapy remotely, both to save time and to enable remote delivery of treatment with minimal therapist support. Due to the limited number of trained therapists, it is recommended that routine and regular training for all practitioners involved in the diagnosis and treatment of TS can effectively help more patients in need.

#### Basal ganglia

The basal ganglia has important motor regulation functions. The abnormality of the basal ganglia and its relationship with the cortical areas is the key to understanding the pathophysiology of TS (Kleimaker et al., 2021). Evidence from a number of studies suggests that impaired function of the cortical-basal ganglia-thalamic-cortical circuit and a dysfunction of many neurotransmitter systems are probably responsible for TS (Kumar et al., 2016; Singer, 2019). Disruption of the cortical-basal ganglia-thalamic-cortical circuit can cause a message to the primary motor cortex to be disrupted. The phonic and motor tics are caused by this. Numerous neurotransmitters are actively utilized in the circuit and involved in the pathophysiology of TS, including dopamine, glutamate, γ-aminobutyric acid (GABA), serotonin, acetylcholine, norepinephrine, cannabinoids, opioids, and histamine (Augustine and Singer, 2018; Nielsen et al., 2020).

#### Pharmacological treatment

In 2021, the European Society for the Study of Tourette Syndrome (ESSTS) published online the latest European guidelines for TS in the *European Journal of Child and Adolescent Psychiatry*, which pointed out that when the curative effect of behavioral therapy is poor or ineffective or unavailable, additional pharmacological treatment should be considered (Rizwan et al., 2022), including antipsychotics, α-agonists, botulinum toxin injections, cannabis-based medications, antiseizure medications, and traditional Chinese medicines (Pringsheim et al., 2019b). The ESSTS recommended aripiprazole, tiapride, and risperidone for TS, in which aripiprazole was considered to be the first choice for children and adults (Roessner et al., 2022). The AAN analyzed the evidence and found that the evidence quality of antipsychotics (haloperidol, risperidone, aripiprazole, tiapride), clonidine, onabotulinum toxin A injections, and traditional Chinese medicine (ningdong granule, 5-ling granule) was higher, which indicated that these interventions are probably more likely than placebo to have reduced tic severity (Pringsheim et al., 2019a). In pharmacologic cases where rapid reduction of tics is urgently required. At present, pharmacological treatment can reduce tics by 60–90%, such as aripiprazole by 74% (Sallee et al., 2017). However, each drug has well-known adverse effects, including weight gain, elevated prolactin levels, sedation, drug-induced movement disorders, and effects on heart rate, blood pressure, and electrocardiograms (Pringsheim et al., 2019a). Therefore, when selecting the most appropriate drug for a patient with TS, the efficacy and potential adverse effects of the drug should be considered, and it is recommended that adverse events be carefully monitored and dosage should be gradually reduced during withdrawal. Meanwhile, another important point is the presence of comorbidities when choosing a drug for a patient. Clonidine and guanfacine for TS and ADHD are the best options (Roessner et al., 2022). Behavioral therapy is the first-line treatment for patients with TS and OCD, followed by selective serotonin reuptake inhibitors (SSRIs). Generally speaking, the treatment of TS should be individualized, and pharmacological treatment should consider all relevant factors. We suggest that patients with TS should be evaluated and examined regularly to evaluate the effectiveness of the drug, any side effects, and the necessity for further therapy.

#### Risk factors

In a logistic regression analysis of 6,090 children in the Avon Longitudinal Study of Parents and Children (ALSPAC), United Kingdom, Carol et al. concluded that primiparity (first-born), maternal alcohol, inadequate maternal weight gain during pregnancy, and cannabis use were the main candidate environmental risk factors for TS (Mathews et al., 2014). Several references have reported that maternal smoking, psychosocial stress during pregnancy, and low birth weight were prenatal risk factors for TS (Chao et al., 2014; Browne et al., 2016; Leckman and Fernandez, 2016; Brander et al., 2018; Ayubi et al., 2021). A large nationwide cohort study with data from the Finnish Medical Birth Register showed nulliparity was associated with increased odds for TS, but birth weight of 4,000–4,499 g was associated with decreased odds for TS (Leivonen et al., 2016). In addition, risk factors for TS also included family history, impaired fetal growth, cesarean section, maternal autoimmune disease, group A streptococcal (GAS), and other infections, and allergic illnesses (Leivonen et al., 2016; Brander et al., 2018; Han et al., 2021). At the same time, risk genes are also worthy of attention. At the moment, genes like FLT3, SLITRK1, HDC, CNTN6, NRXN1, PNKD, KCNJ5, and CELSR3 have been suggested as possible susceptibility genes (Gomez et al., 2014; Singh et al., 2016; Sun et al., 2018; Yu et al., 2019; Set and Warner, 2021), but further research is needed to determine their specific mechanisms. Further understanding of these risk factors will be helpful in the development of interventions and in focusing future research initiatives to reduce the burden associated with TS. These risk factors can be reduced as much as possible through prenatal education, communication between doctors and patients, and careful monitoring of patients who may have the tendency to TS. Consequently, it is advised that all clinicians involved in the treatment of TS should conduct a condition evaluation to understand the risk factors associated with the development of TS. In the future research direction, the risk factors of TS are unique or common.

## Strengths and limitations

To the best of our knowledge, this study is the first to use CiteSpace for bibliometric visual analysis of TS in children. Make a clear visual display of the number of documents, journals, references, and other aspects, interpret the result data from multiple angles, summarize the research status in this field, analyze the research hotspots, and predict the future research trend. At the same time, we downloaded the data from the WoSCC database. The source is relatively comprehensive, and the analysis is more objective. However, this study also has some limitations. First of all, due to the limitations of CiteSpace, we only analyze data from WoSCC, so the published content may not be complete. It is expected that with the improvement of the software functions in the future, a wider range of options can be realized. Secondly, our analysis selects English literature, which makes the analysis results not applicable in some places, because researchers cannot fully understand other languages. Thirdly, in the analysis process, it is found that there are many synonyms, which may cause some overlap in the results of cluster analysis.

## Conclusion

According to the findings of CiteSpace, publications related to TS in children have increased in the past 10 years. The USA has the highest publication rate and the most productive institutions and researchers, but the cooperation among countries/regions, institutions, and authors around the world is still not close enough. Current research hotspots mainly include epidemiology, comorbidities, deep brain stimulation, behavior therapy, and basal ganglia. The main research trends include comorbidities, pharmacological treatment, and risk factors. Therefore, international cooperation should be strengthened in the future, and attention should be devoted to the psychiatric comorbidities of TS, the choice of intervention measures, and early warning of risk factors.

## Data availability statement

Publicly available datasets were analyzed in this study. This data can be found here: the Web of Science Core Collection.

## Author contributions

CY created and analyzed the data. CY and JieZ wrote and edited the manuscript. LW, CY, QZ, JJZ, and JiaZ worked on the manuscript revision. All authors contributed to the manuscript revision and approved the submitted version.
