# Supplementary material for: Trends of Tourette Syndrome in children from 2011 to 2021: A bibliometric analysis
Source: Front Behav Neurosci. 2022 Nov 17;16:991805. doi: 10.3389/fnbeh.2022.991805 (PMC9714309; doi:10.3389/fnbeh.2022.991805)
Supplement: Supplementary file 1 [file Data_Sheet_1.PDF]

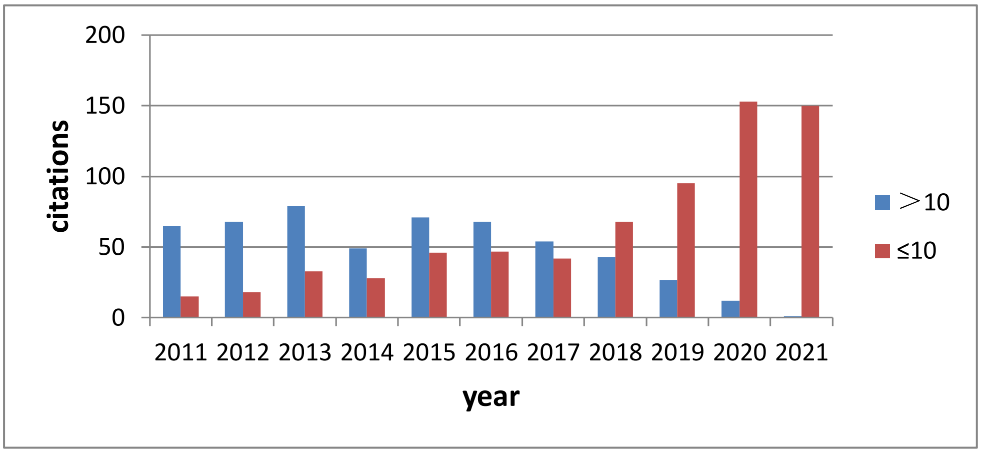

FIG. 1 Trend chart of annual published quantity after grouping citations（2021）.

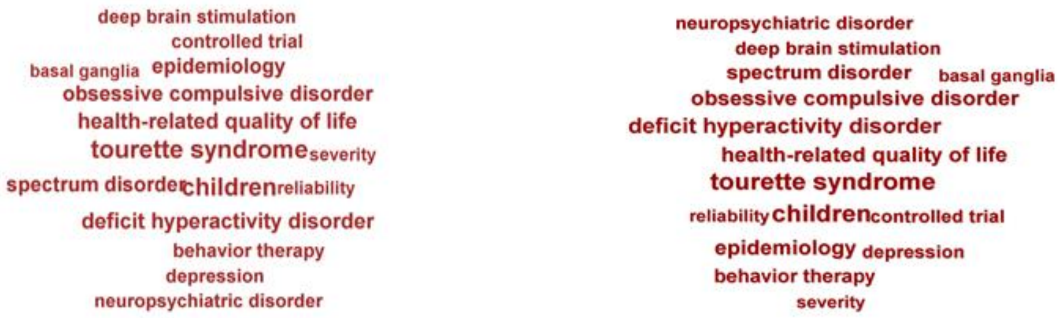

A B

FIG. 2 Keyword atlas of A with influence factor > 10; B Keyword atlas with influence factor ≤10
