# Supplementary material for: Trends of Tourette Syndrome in children from 2011 to 2021: A bibliometric analysis
Source: Front Behav Neurosci. 2022 Nov 17;16:991805. doi: 10.3389/fnbeh.2022.991805 (PMC9714309; doi:10.3389/fnbeh.2022.991805)
Supplement: Supplementary file 2 [file Data_Sheet_2.PDF]

TABLE 1 | Grouping of impact factors (2021)

	IF＞5	IF≤5
Journals	112	284
Articles	467	765

TABLE2 | The number of published articles per year is grouped according to the impact factor (2021)

	IF＞5	IF≤5	All
**2011**	33	47	80
**2012**	31	55	86
**2013**	42	70	112
**2014**	30	47	77
**2015**	40	77	117
**2016**	50	65	115
**2017**	41	55	96
**2018**	48	63	111
**2019**	43	79	122
**2020**	56	109	165
**2021**	53	98	151

TABLE 3 | The distribution of impact factors(2021) and JCR partition for the journals

**Journal**	**Frequency**	IF(2021)	**JCR partition**
BMJ-BRITISH MEDICAL JOURNAL	3	93.333	Q1
LANCET PSYCHIATRY	1	77.056	Q1
NATURE REVIEWS DISEASE PRIMERS	1	65.038	Q1
LANCET NEUROLOGY	1	59.935	Q1
NATURE REVIEWS NEUROLOGY	1	44.711	Q1
LANCET CHILD & ADOLESCENT HEALTH	1	37.746	Q1
MMWR-MORBIDITY AND MORTALITY WEEKLY REPORT	1	35.301	Q1
CELL METABOLISM	1	31.373	Q1
JAMA NEUROLOGY	2	29.907	Q1
JAMA PSYCHIATRY	9	25.911	Q1
PHARMACOLOGY & THERAPEUTICS	1	23.4	Q1
AMERICAN JOURNAL OF PSYCHIATRY	2	19.242	Q1
BRAIN BEHAVIOR AND IMMUNITY	6	19.227	Q1
AUTOIMMUNITY REVIEWS	1	17.39	Q1
ALZHEIMERS & DEMENTIA	1	16.655	Q1
BRAIN	5	15.255	Q1
ARCHIVES OF GENERAL PSYCHIATRY	2	14.48	Q1
ASIAN JOURNAL OF PSYCHIATRY	1	13.89	Q1
JOURNAL OF NEUROLOGY NEUROSURGERY AND PSYCHIATRY	3	13.654	Q1
MOLECULAR PSYCHIATRY	4	13.437	Q1

JAMA NETWORK OPEN	4	13.353	Q3
JOURNAL OF THE AMERICAN ACADEMY OF CHILD AND ADOLESCENT PSYCHIATRY	19	13.113	Q1
BIOLOGICAL PSYCHIATRY	6	12.81	Q1
PROCEEDINGS OF THE NATIONAL ACADEMY OF SCIENCES OF THE UNITED STATES OF AMERICA	1	12.779	Q1
PSYCHIATRY AND CLINICAL NEUROSCIENCES	1	12.145	Q1
COCHRANE DATABASE OF SYSTEMATIC REVIEWS	5	12.008	Q1
NEUROLOGY	8	11.8	Q1
DRUGS	2	11.431	Q1
SLEEP MEDICINE REVIEWS	1	11.401	Q1
PSYCHIATRY RESEARCH	36	11.225	Q1
CURRENT BIOLOGY	2	10.9	Q1
PROGRESS IN NEUROBIOLOGY	1	10.885	Q1
BRITISH JOURNAL OF PSYCHIATRY	4	10.671	Q1
PSYCHOLOGICAL MEDICINE	5	10.592	Q1
CHEST	1	10.262	Q1
TRANSLATIONAL NEURODEGENERATION	1	9.883	Q1
PEDIATRICS	5	9.703	Q1
MOVEMENT DISORDERS	24	9.698	Q1
BRITISH JOURNAL OF PHARMACOLOGY	1	9.473	Q1
BRAIN STIMULATION	1	9.184	Q1
BIOSCIENCE TRENDS	1	9.083	Q1
NEUROSCIENCE AND BIOBEHAVIORAL REVIEWS	17	9.052	Q1
JOURNAL OF HEADACHE AND PAIN	1	8.588	Q1
PATTERN RECOGNITION	1	8.518	Q1
ENVIRONMENTAL RESEARCH	1	8.431	Q1
DEVELOPMENTAL REVIEW	1	8.361	Q1
NEUROPSYCHOPHARMACOLOGY	1	8.294	Q1
JOURNAL OF CHILD PSYCHOLOGY AND PSYCHIATRY	28	8.265	Q1
DEUTSCHES ARZTEBLATT INTERNATIONAL	1	8.251	Q1
DEPRESSION AND ANXIETY	3	8.128	Q1
CURRENT PSYCHIATRY REPORTS	3	8.081	Q1
TRANSLATIONAL PSYCHIATRY	3	7.989	Q1
JOURNAL OF ADOLESCENT HEALTH	1	7.83	Q1
EPIDEMIOLOGY AND PSYCHIATRIC SCIENCES	2	7.818	Q1
ACTA PSYCHIATRICA SCANDINAVICA	2	7.734	Q1
CURRENT NEUROPHARMACOLOGY	1	7.708	Q1
JOURNAL OF ABNORMAL PSYCHOLOGY	1	7.507	Q1
CHILD AND ADOLESCENT PSYCHIATRY AND MENTAL HEALTH	7	7.494	Q1
NEUROIMAGE	1	7.4	Q1
JOURNAL OF NEUROIMMUNE PHARMACOLOGY	1	7.285	Q1
COMPREHENSIVE PSYCHIATRY	15	7.211	Q1
EUROPEAN PSYCHIATRY	3	7.156	Q1

JOURNAL OF MEDICAL INTERNET RESEARCH	4	7.076	Q1
CNS NEUROSCIENCE & THERAPEUTICS	2	7.035	Q1
JOURNAL OF NEUROSCIENCE	2	6.709	Q1
NUTRIENTS	2	6.706	Q1
JOURNAL OF NEUROLOGY	3	6.682	Q1
JOURNAL OF AFFECTIVE DISORDERS	7	6.533	Q1
CHILDHOOD ONSET DEVELOPMENTAL DISORDERS	2	6.499	Q1
CNS DRUGS	4	6.497	Q1
MOLECULAR AUTISM	1	6.476	Q1
HUMAN REPRODUCTION	1	6.353	Q1
JOURNAL OF TELEMEDICINE AND TELECARE	1	6.344	Q1
JMIR MENTAL HEALTH	1	6.332	Q2
REVISTA BRASILEIRA DE PSIQUIATRIA	2	6.328	Q2
INTERNATIONAL JOURNAL OF NEURAL SYSTEMS	1	6.325	Q1
JOURNAL OF PEDIATRICS	6	6.314	Q1
CLINICA CHIMICA ACTA	1	6.314	Q1
SLEEP	2	6.313	Q1
EUROPEAN JOURNAL OF NEUROLOGY	1	6.288	Q1
CURRENT OPINION IN NEUROLOGY	4	6.283	Q1
FRONTIERS IN MOLECULAR NEUROSCIENCE	1	6.261	Q1
NEUROTHERAPEUTICS	2	6.088	Q1
CEPHALALGIA	1	6.075	Q1
FRONTIERS IN MICROBIOLOGY	1	6.064	Q1
CURRENT NEUROLOGY AND NEUROSCIENCE REPORTS	1	6.03	Q1
AMERICAN JOURNAL OF CHINESE MEDICINE	1	6.005	Q1
FRONTIERS IN PHARMACOLOGY	1	5.988	Q1
JOURNAL OF MEDICAL ETHICS	1	5.926	Q1
JOURNAL OF CLINICAL PSYCHIATRY	4	5.906	Q2
CLINICAL EPIDEMIOLOGY	1	5.814	Q1
DEVELOPMENTAL COGNITIVE NEUROSCIENCE	5	5.811	Q1
EUROPEAN ARCHIVES OF PSYCHIATRY AND CLINICAL NEUROSCIENCE	2	5.76	Q2
MOLECULAR NEUROBIOLOGY	2	5.682	Q1
AUSTRALIAN AND NEW ZEALAND JOURNAL OF PSYCHIATRY	1	5.598	Q2
FRONTIERS IN PSYCHIATRY	31	5.435	Q2
EUROPEAN NEUROPSYCHOPHARMACOLOGY	1	5.415	Q1
HUMAN BRAIN MAPPING	3	5.399	Q1
NEUROEPIDEMIOLOGY	2	5.393	Q1
EUROPEAN JOURNAL OF HUMAN GENETICS	1	5.351	Q2
EUROPEAN CHILD & ADOLESCENT PSYCHIATRY	48	5.349	Q1
CANADIAN JOURNAL OF PSYCHIATRY-REVUE CANADIENNE DE PSYCHIATRIE	3	5.321	Q2
INDIAN JOURNAL OF PEDIATRICS	1	5.319	Q1
AMERICAN FAMILY PHYSICIAN	2	5.305	Q2

NEUROPHARMACOLOGY	1	5.273	Q2
JOURNAL OF PSYCHIATRIC RESEARCH	14	5.25	Q2
PROGRESS IN NEURO-PSYCHOPHARMACOLOGY & BIOLOGICAL PSYCHIATRY	3	5.201	Q2
CHEMICO-BIOLOGICAL INTERACTIONS	1	5.168	Q2
BJPSYCH OPEN	2	5.165	Q2
FRONTIERS IN NEUROSCIENCE	10	5.152	Q2
APPETITE	2	5.016	Q3
JOURNAL OF STEROID BIOCHEMISTRY AND MOLECULAR BIOLOGY	1	5.011	Q2
SCIENTIFIC REPORTS	7	4.996	Q2
THERAPEUTIC ADVANCES IN PSYCHOPHARMACOLOGY	2	4.988	Q2
POSTGRADUATE MEDICAL JOURNAL	1	4.973	Q2
JOURNAL OF CLINICAL MEDICINE	2	4.964	Q2
ARCHIVES OF DISEASE IN CHILDHOOD	5	4.92	Q1
JOURNAL OF EATING DISORDERS	1	4.916	Q2
NEUROIMAGE-CLINICAL	3	4.891	Q2
DEVELOPMENTAL MEDICINE AND CHILD NEUROLOGY	16	4.864	Q2
CHILD ABUSE & NEGLECT	2	4.863	Q1
CLINICAL NEUROPHYSIOLOGY	2	4.861	Q2
SLEEP MEDICINE	3	4.842	Q2
BEHAVIOR THERAPY	1	4.796	Q2
CURRENT OPINION IN PSYCHIATRY	2	4.787	Q2
NEUROPSYCHIATRY	4	4.778	Q1
IEEE TRANSACTIONS ON BIOMEDICAL ENGINEERING	1	4.756	Q2
REVIEWS IN THE NEUROSCIENCES	1	4.703	Q2
PSYCHONEUROENDOCRINOLOGY	1	4.693	Q2
CORTEX	12	4.644	Q2
AUTISM RESEARCH	1	4.633	Q1
JOURNAL OF PSYCHOSOMATIC RESEARCH	2	4.62	Q2
DISABILITY AND HEALTH JOURNAL	1	4.615	Q1
INTERNATIONAL JOURNAL OF ENVIRONMENTAL RESEARCH AND PUBLIC HEALTH	5	4.614	Q2
CNS SPECTRUMS	5	4.604	Q2
ADDICTIVE BEHAVIORS	1	4.591	Q2
JOURNAL OF PSYCHOPHARMACOLOGY	2	4.562	Q2
CONFLICT AND HEALTH	1	4.554	Q2
JOURNAL OF THE NEUROLOGICAL SCIENCES	1	4.553	Q2
CHINESE MEDICINE	1	4.546	Q1
CHILDS NERVOUS SYSTEM	7	4.532	Q4
EUROPEAN JOURNAL OF RADIOLOGY	1	4.531	Q3
SOCIAL PSYCHIATRY AND PSYCHIATRIC EPIDEMIOLOGY	1	4.519	Q2
MOVEMENT DISORDERS CLINICAL PRACTICE	1	4.514	Q2
ACTA NEUROPSYCHIATRICA	1	4.513	Q2

INTEGRATIVE MEDICINE RESEARCH	1	4.473	Q1
NEUROLOGY AND THERAPY	1	4.446	Q2
JOURNAL OF CLINICAL NURSING	2	4.423	Q1
PSYCHOPHARMACOLOGY	1	4.415	Q2
NEUROCHEMICAL RESEARCH	2	4.414	Q2
PARKINSONISM & RELATED DISORDERS	3	4.402	Q2
MOLECULAR BRAIN	2	4.399	Q2
POSTGRADUATE MEDICINE	1	4.379	Q2
REVUE NEUROLOGIQUE	1	4.313	Q2
EXPERT REVIEW OF NEUROTHERAPEUTICS	6	4.287	Q2
ADVANCES IN THE NEUROCHEMISTRY AND NEUROPHARMACOLOGY OF TOURETTE SYNDROME	3	4.28	Q2
IMAGING IN MOVEMENT DISORDERS: IMAGING APPLICATIONS IN NON-PARKINSONIAN AND OTHER MOVEMENT DISORDERS	1	4.28	Q2
BRAIN TOPOGRAPHY	2	4.275	Q2
QUALITATIVE HEALTH RESEARCH	1	4.233	Q2
FRONTIERS IN PSYCHOLOGY	2	4.232	Q1
DEMOGRAPHY	1	4.222	Q1
PEDIATRIC NEUROLOGY	20	4.21	Q2
VACCINE	1	4.169	Q3
PSYCHIATRIC SERVICES	1	4.157	Q2
BMC PSYCHIATRY	15	4.144	Q2
BIOCHIMICA ET BIOPHYSICA ACTA-GENERAL SUBJECTS	1	4.117	Q3
CHILD AND ADOLESCENT MENTAL HEALTH	3	4.111	Q1
HEALTH TECHNOLOGY ASSESSMENT	1	4.106	Q2
EXPERT OPINION ON PHARMACOTHERAPY	1	4.103	Q2
PEDIATRIC PULMONOLOGY	2	4.09	Q1
FRONTIERS IN NEUROLOGY	17	4.086	Q2
JOURNAL OF NEURODEVELOPMENTAL DISORDERS	2	4.074	Q2
NEUROTOXICOLOGY AND TERATOLOGY	1	4.071	Q2
ACTA PAEDIATRICA	3	4.056	Q1
TRANSLATIONAL PEDIATRICS	2	4.047	Q1
NEUROTOXICITY RESEARCH	2	3.978	Q2
BIOSCIENCE REPORTS	1	3.976	Q3
CURRENT TREATMENT OPTIONS IN NEUROLOGY	2	3.972	Q2
PEDIATRIC RESEARCH	2	3.953	Q1
BEHAVIORAL AND BRAIN FUNCTIONS	1	3.95	Q1
PEDIATRIC DRUGS	1	3.93	Q1
CYTOKINE	1	3.926	Q3
PEDIATRIC OBESITY	1	3.91	Q1
HARVARD REVIEW OF PSYCHIATRY	1	3.868	Q3
PSYCHOSOMATIC MEDICINE	1	3.864	Q3
MUSCLE & NERVE	1	3.852	Q2
JOURNAL OF NEURAL TRANSMISSION	7	3.85	Q2

INDIAN PEDIATRICS	1	3.839	Q1
NEUROLOGICAL SCIENCES	8	3.83	Q2
PEDIATRIC INFECTIOUS DISEASE JOURNAL	2	3.806	Q3
EUROPEAN REVIEW FOR MEDICAL AND PHARMACOLOGICAL SCIENCES	2	3.78	Q2
ASIA-PACIFIC PSYCHIATRY	1	3.778	Q3
PLOS ONE	12	3.752	Q2
PHYSIOLOGY & BEHAVIOR	1	3.742	Q1
CLINICAL PSYCHOPHARMACOLOGY AND NEUROSCIENCE	2	3.731	Q3
GENES BRAIN AND BEHAVIOR	2	3.708	Q2
NEUROSCIENCE	1	3.708	Q3
EUROPEAN JOURNAL OF NEUROSCIENCE	1	3.698	Q3
EUROPEAN JOURNAL OF PAEDIATRIC NEUROLOGY	13	3.692	Q2
CEREBELLUM	1	3.648	Q3
BMC MEDICAL GENOMICS	1	3.622	Q2
FRONTIERS IN BEHAVIORAL NEUROSCIENCE	1	3.617	Q2
ANNALS OF TRANSLATIONAL MEDICINE	1	3.616	Q3
APPLIED ACOUSTICS	1	3.614	Q1
BRAIN RESEARCH	1	3.61	Q3
PEDIATRIC CLINICS OF NORTH AMERICA	3	3.58	Q2
CLINICAL DRUG INVESTIGATION	1	3.58	Q3
BALKAN MEDICAL JOURNAL	1	3.57	Q2
FRONTIERS IN PEDIATRICS	7	3.569	Q2
DNA AND CELL BIOLOGY	1	3.55	Q3
GRAEFES ARCHIVE FOR CLINICAL AND EXPERIMENTAL OPHTHALMOLOGY	1	3.535	Q2
AGING & MENTAL HEALTH	1	3.514	Q3
JOURNAL OF PERSONALIZED MEDICINE	1	3.508	Q2
PSYCHIATRIC QUARTERLY	1	3.504	Q2
WORLD JOURNAL OF PSYCHIATRY	1	3.5	Q3
JOURNAL OF PROSTHODONTICS-IMPLANT ESTHETIC AND RECONSTRUCTIVE DENTISTRY	1	3.485	Q2
FRONTIERS IN HUMAN NEUROSCIENCE	7	3.473	Q3
QUALITY OF LIFE RESEARCH	1	3.44	Q2
NEUROPSYCHOLOGY	2	3.424	Q3
WORLD JOURNAL OF BIOLOGICAL PSYCHIATRY	4	3.418	Q3
NEURODEGENERATIVE DISEASES	1	3.417	Q3
SEIZURE-EUROPEAN JOURNAL OF EPILEPSY	3	3.414	Q3
MEDICAL SCIENCE MONITOR	1	3.386	Q3
NATURE AND SCIENCE OF SLEEP	1	3.384	Q3
AMERICAN JOURNAL OF MEDICAL GENETICS PART C-SEMINARS IN MEDICAL GENETICS	3	3.359	Q3
AMERICAN JOURNAL OF MEDICAL GENETICS PART B-NEUROPSYCHIATRIC GENETICS	6	3.358	Q3

BEHAVIOURAL BRAIN RESEARCH	2	3.352	Q3
COMPLEMENTARY THERAPIES IN MEDICINE	1	3.335	Q2
BRAIN SCIENCES	7	3.333	Q3
ANNALS OF GENERAL PSYCHIATRY	4	3.301	Q3
INTERNATIONAL JOURNAL OF IMMUNOPATHOLOGY AND PHARMACOLOGY	2	3.298	Q3
ITALIAN JOURNAL OF PEDIATRICS	2	3.288	Q2
BMC NEUROSCIENCE	2	3.264	Q3
BIOMED RESEARCH INTERNATIONAL	1	3.246	Q3
JOURNAL OF INVESTIGATIVE MEDICINE	1	3.235	Q2
DISCOVERY MEDICINE	1	3.222	Q3
JOURNAL OF NEUROIMMUNOLOGY	3	3.221	Q4
PSYCHIATRY INVESTIGATION	2	3.202	Q3
NEUROSCIENCE LETTERS	5	3.197	Q3
JOURNAL OF ATTENTION DISORDERS	13	3.196	Q3
NEURAL PLASTICITY	1	3.144	Q3
ARCHIVES OF IRANIAN MEDICINE	2	3.138	Q2
JOURNAL OF CLINICAL LABORATORY ANALYSIS	1	3.124	Q2
JOURNAL OF CLINICAL PSYCHOPHARMACOLOGY	4	3.118	Q3
JOURNAL OF THE INTERNATIONAL NEUROPSYCHOLOGICAL SOCIETY	1	3.114	Q3
BEHAVIOURAL NEUROLOGY	13	3.112	Q3
PAEDIATRIC AND PERINATAL EPIDEMIOLOGY	1	3.103	Q2
EUROPEAN JOURNAL OF CLINICAL PHARMACOLOGY	3	3.064	Q3
PEERJ	2	3.061	Q2
JOURNAL OF ADVANCED NURSING	1	3.057	Q1
NEUROPSYCHOLOGIA	2	3.054	Q3
SEMINARS IN PEDIATRIC NEUROLOGY	5	3.042	Q3
JOURNAL OF CHILD AND ADOLESCENT PSYCHOPHARMACOLOGY	47	3.031	Q2
CANADIAN FAMILY PHYSICIAN	1	3.025	Q2
FUTURE VIROLOGY	1	3.015	Q3
CONTRAST MEDIA & MOLECULAR IMAGING	1	3.009	Q3
BMJ OPEN	5	3.006	Q2
JOURNAL OF THORACIC DISEASE	2	3.005	Q3
ACADEMIC PEDIATRICS	2	2.993	Q2
NEUROPSYCHIATRIC DISEASE AND TREATMENT	10	2.989	Q3
JOURNAL OF DEVELOPMENTAL AND BEHAVIORAL PEDIATRICS	13	2.988	Q3
JOURNAL OF NEUROSCIENCE METHODS	2	2.987	Q3
INDIAN JOURNAL OF PSYCHIATRY	2	2.983	Q3
JOURNAL OF NEUROPHYSIOLOGY	1	2.974	Q3
CHILD CARE HEALTH AND DEVELOPMENT	2	2.943	Q2
CANADIAN JOURNAL OF NEUROLOGICAL SCIENCES	2	2.915	Q3
BMC NEUROLOGY	2	2.903	Q3
CURRENT OPINION IN PEDIATRICS	1	2.893	Q2

JOURNAL OF NEUROPSYCHIATRY AND CLINICAL NEUROSCIENCES	7	2.891	Q3
JOURNAL OF MOLECULAR NEUROSCIENCE	1	2.866	Q4
CHILDREN-BASEL	2	2.835	Q2
ACTA NEUROCHIRURGICA	1	2.816	Q3
BRAIN AND LANGUAGE	1	2.781	Q2
CHILD PSYCHIATRY & HUMAN DEVELOPMENT	7	2.776	Q3
EXPERIMENTAL AND THERAPEUTIC MEDICINE	1	2.751	Q4
ENTROPY	1	2.738	Q2
TRIALS	4	2.728	Q4
JOURNAL OF NEUROSURGERY-PEDIATRICS	1	2.713	Q3
CURRENT MEDICAL RESEARCH AND OPINION	1	2.705	Q3
EARLY HUMAN DEVELOPMENT	1	2.699	Q3
PSYCHIATRIA DANUBINA	1	2.696	Q3
BEHAVIOR MODIFICATION	2	2.692	Q3
ANNALS OF CLINICAL PSYCHIATRY	1	2.691	Q4
BRAIN AND COGNITION	1	2.682	Q4
JOURNAL OF BEHAVIOR THERAPY AND EXPERIMENTAL PSYCHIATRY	1	2.662	Q3
EVIDENCE-BASED COMPLEMENTARY AND ALTERNATIVE MEDICINE	2	2.65	Q3
BURNS	1	2.609	Q3
PAEDIATRICS & CHILD HEALTH	1	2.6	Q3
CHILD NEUROPSYCHOLOGY	4	2.597	Q3
INTERNATIONAL JOURNAL OF NEUROSCIENCE	1	2.59	Q4
PEDIATRICS AND NEONATOLOGY	2	2.586	Q3
AMERICAN JOURNAL OF MEDICAL GENETICS PART A	2	2.578	Q3
AMERICAN JOURNAL OF NURSING	1	2.577	Q2
PSYCHIATRIC GENETICS	2	2.574	Q3
BMC PEDIATRICS	4	2.567	Q3
CURRENT PROBLEMS IN PEDIATRIC AND ADOLESCENT HEALTH CARE	2	2.557	Q3
COGNITIVE NEUROSCIENCE	1	2.55	Q4
JOURNAL OF TRADITIONAL CHINESE MEDICINE	1	2.547	Q3
PHARMACOPSYCHIATRY	1	2.544	Q3
INTERNATIONAL JOURNAL OF DEVELOPMENTAL NEUROSCIENCE	3	2.54	Q3
JOURNAL OF FLUORESCENCE	1	2.525	Q4
JOURNAL OF PEDIATRIC NURSING-NURSING CARE OF CHILDREN & FAMILIES	1	2.523	Q2
PSYCHIATRY RESEARCH-NEUROIMAGING	5	2.493	Q3
MOLECULAR GENETICS & GENOMIC MEDICINE	1	2.473	Q3
ACTA NEUROLOGICA BELGICA	1	2.471	Q3
TWIN RESEARCH AND HUMAN GENETICS	1	2.47	Q3
COMMUNITY MENTAL HEALTH JOURNAL	1	2.469	Q3

IN VIVO	1	2.406	Q4
CURRENT PSYCHOLOGY	1	2.387	Q3
INTERNATIONAL JOURNAL OF IMMUNOGENETICS	1	2.385	Q3
SOCIAL NEUROSCIENCE	1	2.381	Q4
JOURNAL OF CHILD NEUROLOGY	32	2.363	Q3
APPLIED COGNITIVE PSYCHOLOGY	1	2.36	Q3
PAKISTAN JOURNAL OF MEDICAL SCIENCES	2	2.34	Q3
CIRCUITS SYSTEMS AND SIGNAL PROCESSING	1	2.311	Q3
JOURNAL OF VOICE	1	2.3	Q2
BIOLOGICAL JOURNAL OF THE LINNEAN SOCIETY	1	2.277	Q4
JOURNAL OF NEUROPSYCHOLOGY	8	2.276	Q3
BRAIN & DEVELOPMENT	11	2.272	Q2
CONTEMPORARY CLINICAL TRIALS	1	2.261	Q4
ARCHIVES OF PSYCHIATRIC NURSING	1	2.242	Q2
JOURNAL OF OBSESSIVE-COMPULSIVE AND RELATED DISORDERS	17	2.236	Q4
NEUROLOGIA I NEUROCHIRURGIA POLSKA	3	2.223	Q4
INDIAN JOURNAL OF DERMATOLOGY VENEREOLOGY & LEPROLOGY	1	2.217	Q3
HUMAN PSYCHOPHARMACOLOGY-CLINICAL AND EXPERIMENTAL	3	2.13	Q4
JOURNAL OF CLINICAL NEUROSCIENCE	2	2.116	Q4
NORDIC JOURNAL OF PSYCHIATRY	7	2.099	Q4
ALLERGOLOGIA ET IMMUNOPATHOLOGIA	1	2.094	Q4
CLINICAL CHILD PSYCHOLOGY AND PSYCHIATRY	1	2.087	Q4
AUSTRALIAN JOURNAL OF GENERAL PRACTICE	1	2.071	Q3
EXPERIMENTAL BRAIN RESEARCH	1	2.064	Q4
CLINICAL EEG AND NEUROSCIENCE	1	2.046	Q4
JOURNAL OF COMPARATIVE EFFECTIVENESS RESEARCH	1	2.04	Q4
EXPERT REVIEW OF PHARMACOECONOMICS & OUTCOMES RESEARCH	1	2.039	Q4
ARQUIVOS DE NEURO-PSIQUIATRIA	2	2.035	Q4
INTERNATIONAL OPHTHALMOLOGY	1	2.029	Q3
INTERNATIONAL CLINICAL PSYCHOPHARMACOLOGY	2	2.023	Q4
BMC MEDICAL GENETICS	1	2.023	Q4
COGNITIVE NEUROPSYCHIATRY	2	1.957	Q4
JOURNAL OF PAEDIATRICS AND CHILD HEALTH	2	1.929	Q3
JOURNAL OF PEDIATRIC UROLOGY	1	1.921	Q3
FEMALE PELVIC MEDICINE AND RECONSTRUCTIVE SURGERY	1	1.913	Q4
JOURNAL OF NERVOUS AND MENTAL DISEASE	1	1.899	Q4
JOURNAL OF CHILD HEALTH CARE	1	1.896	Q3
CLINICAL NEUROLOGY AND NEUROSURGERY	1	1.885	Q4
DANISH MEDICAL JOURNAL	1	1.865	Q3
JOURNAL OF PEDIATRIC HEALTH CARE	1	1.838	Q4
AUSTRALASIAN PSYCHIATRY	1	1.837	Q4

MEDICINE	6	1.817	Q3
AUSTRALIAN OCCUPATIONAL THERAPY JOURNAL	1	1.757	Q3
GENETIC TESTING AND MOLECULAR BIOMARKERS	1	1.736	Q4
ARTS & HEALTH	1	1.724	Q4
CLINICAL PEDIATRICS	2	1.701	Q4
NEUROPEDIATRICS	2	1.696	Q4
ACTAS ESPANOLAS DE PSIQUIATRIA	1	1.667	Q4
NEUROLOGY INDIA	2	1.663	Q4
INTERNATIONAL JOURNAL OF PEDIATRIC OTORHINOLARYNGOLOGY	2	1.626	Q4
PEDIATRICS INTERNATIONAL	1	1.617	Q4
APPLIED NEUROPSYCHOLOGY-CHILD	5	1.613	Q4
PEDIATRIC EMERGENCY CARE	1	1.602	Q3
PSYCHIATRIA POLSKA	1	1.596	Q4
COGNITIVE AND BEHAVIORAL NEUROLOGY	1	1.59	Q4
JOURNAL OF INTERNATIONAL MEDICAL RESEARCH	1	1.573	Q4
TOXICOLOGICAL AND ENVIRONMENTAL CHEMISTRY	1	1.565	Q4
WORLD JOURNAL OF CLINICAL CASES	2	1.534	Q4
IRANIAN JOURNAL OF PUBLIC HEALTH	1	1.479	Q4
NEUROETHICS	2	1.427	Q3
JOVE-JOURNAL OF VISUALIZED EXPERIMENTS	1	1.424	Q3
CLINICAL NEUROPHARMACOLOGY	5	1.379	Q4
JOURNAL OF CONTEMPORARY ETHNOGRAPHY	1	1.368	Q3
PSYCHIATRY AND CLINICAL PSYCHOPHARMACOLOGY	1	1.36	Q4
JOURNAL OF PEDIATRIC OPHTHALMOLOGY & STRABISMUS	2	1.33	Q4
JOURNAL OF AAPOS	1	1.325	Q4
MINERVA PEDIATRICA	1	1.312	Q4
BRITISH JOURNAL OF HOSPITAL MEDICINE	1	1.286	Q4
THESCIENTIFICWORLDJOURNAL	1	1.219	Q2
SCIENTIFIC WORLD JOURNAL	1	1.219	Q2
ARCHIVES OF DISEASE IN CHILDHOOD-EDUCATION AND PRACTICE EDITION	2	1.167	Q4
PEDIATRIC NEUROSURGERY	2	1.165	Q4
SOMATOSENSORY AND MOTOR RESEARCH	1	1.126	Q4
CHILD & FAMILY BEHAVIOR THERAPY	1	1.08	Q4
NOROPSIKIYATRI ARSIVI-ARCHIVES OF NEUROPSYCHIATRY	2	1.066	Q4
JOURNAL OF TRAUMA NURSING	1	0.915	Q4
JOURNAL OF COGNITIVE PSYCHOTHERAPY	1	0.813	Q4
NEUROSCIENCES	2	0.735	Q4
NUTRITION CLINIQUE ET METABOLISME	1	0.704	Q4
ANADOLU PSIKIYATRI DERGISI-ANATOLIAN JOURNAL OF PSYCHIATRY	2	0.658	Q4
KLINIK PSIKOFARMAKOLOJI BULTENI-BULLETIN OF CLINICAL PSYCHOPHARMACOLOGY	3	0.556	Q4

ACTA POLONIAE PHARMACEUTICA	1	0.555	Q4
ZEITSCHRIFT FUR NEUROPSYCHOLOGIE	1	0.553	Q4
NEUROLOGICAL SCIENCES AND NEUROPHYSIOLOGY	1	0.414	Q4
CESKA A SLOVENSKA NEUROLOGIE A NEUROCHIRURGIE	1	0.411	Q4
LIFE SCIENCE JOURNAL-ACTA ZHENGZHOU UNIVERSITY OVERSEAS EDITION	1	0.165	Q4
INTERNATIONAL JOURNAL OF CLINICAL AND EXPERIMENTAL MEDICINE	3	0	-
INTERNATIONAL JOURNAL OF CLINICAL AND EXPERIMENTAL PATHOLOGY	1	0	-
FUNCTIONAL NEUROLOGY	1	0	-

TABLE 4 | Grouping of JCR partitions(2021) *

	Q1	Q2	Q3	Q4
Journals	116	103	103	71
Articles	421	392	281	133

*Three journals (5 articles) were excluded from SCI without impact factors and partitions.

TABLE 5 | The top 10 high-frequency keywords of the two groups

ranking	IF>5		IF≤5	
	Frequency	Keyword	Frequency	Keyword
1	354	tourette syndrome	445	tourette syndrome
2	312	children	421	children
3	135	deficit hyperactivity disorder	165	deficit hyperactivity disorder
4	124	health-related quality of life	150	health-related quality of life
5	118	obsessive compulsive disorder	141	obsessive compulsive disorder
6	107	epidemiology	133	epidemiology
7	103	spectrum disorder	119	spectrum disorder
8	78	behavior therapy	78	controlled trial
9	74	controlled trial	70	depression
10	54	depression	67	behavior therapy

TABLE 6 | Statistics of national publications

ranking	2019	2020	2021	All
1	USA	USA	USA	USA
2	ENGLAND	ENGLAND	GERMANY	ENGLAND.
3	PEOPLES R CHINA	CANADA	ENGLAND	ITALY.
4	SWEDEN	GERMANY	PEOPLES R CHINA	GERMANY.
5	ITALY	SWEDEN	CANADA	PEOPLES R CHINA.
6	CANADA	ITALY	SWEDEN	SWEDEN.
7	GERMANY	PEOPLES R CHINA	ITALY	CANADA.
8	DENMARK	NETHERLANDS	NETHERLANDS	DENMARK.
9	NETHERLANDS	TAIWAN	FRANCE	NETHERLANDS.
10	AUSTRALIA	JAPAN	AUSTRALIA	AUSTRALIA.
